# The Impact of Foundation Medicine Testing on Cancer Patients: A Single Academic Centre Experience

**DOI:** 10.3389/fonc.2021.687730

**Published:** 2021-07-26

**Authors:** Dalia Karol, Mathieu McKinnon, Lenah Mukhtar, Arif Awan, Bryan Lo, Paul Wheatley-Price

**Affiliations:** ^1^ Faculty of Medicine, University of Ottawa, Ottawa, ON, Canada; ^2^ Department of Medicine, Division of Medical Oncology, The Ottawa Hospital Cancer Centre, Ottawa, ON, Canada; ^3^ The Ottawa Hospital Research Institute, Ottawa, ON, Canada; ^4^ Department of Anatomical Pathology, The Ottawa Hospital, Ottawa, ON, Canada

**Keywords:** precision medicine, next-generation sequencing, medical oncology, mutation, cancer, Foundation Medicine

## Abstract

**Background:**

The use of Next-Generation Sequencing (NGS) has recently allowed significant improvements in cancer treatment. Foundation Medicine^®^ (FM) provides a genomic profiling test based on NGS for a variety of cancers. However, it is unclear if the Foundation Medicine test would result in a better outcome than the standard on-site molecular testing. In this retrospective chart review, we identified the FM cases from an academic Canadian hospital and determined whether these test results improved treatment options for those patients.

**Materials and Methods:**

A retrospective analysis was performed on patients with solid tumors who had FM testing between May 1, 2014 and May 1, 2018. Clinical factors and outcomes were measured using descriptive statistics using Microsoft Excel^®^ Software.

**Results:**

Out of 66 FM tests, eight patients (= 12%) had a direct change in therapy based on the FM tests. Identified were 285 oncogenic mutations (median 1, range 0–31); where TP53 (n = 31, 10.9%), CDKN2A (n = 19, 6.7%), KRAS (n = 16, 5.6%) and APC (n = 9, 3.2%) were the most common FM mutations identified.

**Conclusion:**

A small proportion of FM reports identified actionable mutations and led to direct treatment change. FM testing is expensive and a few of the identified mutations are now part of routine on-site testing. NGS testing is likely to become more widespread, but this research suggests that its true clinical impact may be restricted to a minority of patients.

## Introduction

Precision medicine, also known as targeted medicine/genomic medicine or personalized medicine, is increasingly important in the management of cancers (1). Some cancers harbor an ‘oncogenic driver’ mutation, which is a specific mutation that is primarily responsible for the phenotypic expression and growth of that cancer, and offers an opportunity for a targeted treatment that focuses on that specific mutation (2). There are now many examples of such incidences in cancer care in 2020, such as targeting BRAF mutations in melanoma, or numerous driver mutations in lung adenocarcinoma such as EGFR, ALK or ROS1 (3). In 2018, only 8–9% of patients in the US were eligible for genomic-driven anti-cancer treatment with a median response rate of 54% and duration of response of 30 months which is higher than traditional chemotherapy (4). However, the number of druggable alterations in oncology in the near future is projected to dramatically increase to over 40% for cancers such as Non-Small Cell Lung Cancer (NSCLC), bladder, colorectal, breast and melanoma (5).

Some of the significant challenges in the implementation of precision medicine pertain to molecular testing and drug access. With regards to detecting molecular alterations, these can be performed for a few specific oncogenic driver mutations that have an approved and available therapy. However, each test utilizes precious pathological specimen, and with an increasing number of mutations to look for this brings obvious challenges. Furthermore, more clinical trials are selecting patients based on new biomarkers as they have a higher likelihood of success (6). Alternatively, multiple mutations can be identified using next-generation sequencing (NGS), which involves searching for hundreds of possible mutations. However, few of these may have a matching treatment, and indeed those treatments may be unavailable due to regulatory restrictions or delays, or those treatments may still be under investigation (7).Therefore, searching for mutations beyond those that are easily treatable can be a double-edged sword. More information for the patient and clinician may help with prognostication, identification of a clinical trial or even a rare but available therapy (7). Conversely these tests can be expensive, not yield useful information, or potentially yield a useful answer that is then unable to be acted on (7).

Many panels for NGS testing have been developed, and among the first widely available was through the Foundation Medicine^®^ (Cambridge, Massachusetts). Foundation Medicine^®^ testing has been broadly used especially in the USA with FDA approval and in research development to aid with emerging and potential targeted therapies (8, 9). FoundationOne CDX^®^ is a tissue-based test available to Canadians through private pay, with the test costing $4699.63 CAD at the time this manuscript was prepared.

There are many published studies demonstrating that the Foundation Medicine^®^ testing identifies additional mutation information than on-site testing (10–12), with different possible treatments (13, 14). However, there is a lack of evidence on how often Foundation Medicine^®^ yields additional results that actually lead to feasible new treatment options and improved patient outcomes. The cost-effectiveness of NGS has yet to be established although upfront panel testing is less costly than sequential testing (15).

The Ottawa Hospital Cancer Centre is the sole provider of oncology services to a population of 1.4 million patients in Eastern Ontario, Canada. The Ottawa Hospital Molecular Laboratory facilitates requests for these patients to have Foundation Medicine^®^ testing, by reviewing tumor blocks for suitability for testing and selecting the appropriate specimens to be sent. This retrospective chart review explores the cases sent for Foundation Medicine^®^ testing, and describes the mutations discovered, whether they had already been identified locally, and whether FoundationOne CDX^®^ results had any impact in the real-world setting on the ultimate treatment options for the patients.

## Methods

With local research ethics board approval, we completed a single-institution retrospective chart review of all solid-tumor cancer cases that were sent for Foundation Medicine^®^ tissue testing. All histologically confirmed solid tumor cancer cases that had a tumor block sent by the Ottawa Hospital Molecular Lab to Foundation Medicine^®^ between May 1, 2014 and May 1, 2018 were included in this study. For the included cases, data was collected from the patients Foundation Medicine^®^ reports, as well as hospital charts. The data points collected related to patient demographics, their cancer diagnosis and treatments, and results from the Foundation Medicine^®^ reports. This data was collected and stored in a centralized anonymized datasheet.

The primary endpoint for data analysis was the proportion of patients whose Foundation Medicine^®^ report directly impacted the treatment received for that patient, as defined by administration of a therapy directly associated with the Foundation Medicine^®^ test result, and which otherwise would not have been administered. The secondary endpoint for data analysis was a descriptive analysis of the mutations discovered and suitability of samples (were all samples sent ultimately sufficient for analysis). The data was analyzed using descriptive statistics. All statistical analyses were completed using Microsoft Excel software.

## Results

### Demographics

In total, 76 patients had Foundation Medicine^®^ testing performed in the period under study. Of the 76 tests, 10 were excluded due to sample testing failure, leading to 66 or 87% valid patient tests. The reason given by Foundation Medicine^®^ for sample failure were as follows: two were because DNA yield was too low, two were because gross sample blocks were insufficient, five were because there was limited tumor cellularity and one had no details provided by Foundation Medicine^®^. These sample types are as follows: one was a bone curettage, two were endobronchial lung biopsies, two were fine needle aspiration biopsies, three were liver core biopsies, one was a liver segmental biopsy and one was a soft tissue core biopsy.

Of the 66 patients that were included for statistical analysis, the median age at time of cancer diagnosis was 56 years (range 19–84). Twenty-seven (41%) patients were male and 39 (59%) female. At time of Foundation Medicine^®^ testing, all patients were either stage III (15%) or stage IV (85%). The median number of lines of systemic therapy administered prior to Foundation Medicine^®^ testing was 1 (n = 28, 42.4%; range 0–9). The median time from diagnosis to Foundation Medicine^®^ testing was 550 days (range 21–5,531 days). A summary of demographic data can be found in **Table 1**.

**Table 1:** Patient demographic data.

**Age**	
Median (min, max)	56 years (19, 84)
**Sex**	
Male	27 (40.9%)
Female	39 (59.1%)
**Stage at diagnosis**	
I	6 (9.1%)
II	7 (10.6%)
III	17 (25.8%)
IV	36 (54.5%)
**Stage at time of Foundation Medicine ^®^ testing**	
I	0
II	0
III	10 (15.2%)
IV	56 (84.8%)
**Number of lines of therapy prior to Foundation Medicine ^®^ testing**	
0	7 (10.6%)
1	28 (42.4%)
2	15 (22.7%)
3	10 (15.2%)
4 +	6 (9.1%)
**Time from diagnosis to Foundation Medicine ^®^ testing**	
Median (min, max)	550 days (21–5,531)

### Tumor Primary Site and Histology

The most common histological types of cancer identified were: Lung Adenocarcinoma (n = 25, 37.9%), Colon Adenocarcinoma (n = 6, 7.6%) and Breast Ductal Carcinoma (n = 3, 4.6%). A summary of histological types identified are listed in **Table 2**.

**Table 2:** Summary of identified histological types.

Histological types	Number of incidences	Percentage
Adenocarcinoma	43	65.2%
Squamous Cell Carcinoma	4	6.1%
Ductal Cell Carcinoma	3	4.6%
Glioblastoma	2	3.0%
Astrocytoma	1	1.5%
Epithelioid Angiosarcoma	1	1.5%
Leiomyosarcoma	1	1.5%
Lobular Carcinoma	1	1.5%
Melanoma	1	1.5%
Myxoid Liposarcoma	1	1.5%
Neuroectodermal	1	1.5%
Osteosarcoma	1	1.5%
Papillary Carcinoma Tall Cell Variant	1	1.5%
Papillary Urothelial Transitional Cell Carcinoma	1	1.5%
Poorly Differentiated Carcinoma	1	1.5%
Unspecified Sarcoma	1	1.5%
Stromal Sarcoma	1	1.5%

The most common anatomical primary sites of cancer identified were: Lung (n = 27, 40.9%), Colon (n = 6, 9.1%) and Breast (n = 5, 7.6%). A summary of anatomical primary sites identified are listed in **Table 3**.

**Table 3:** Summary of primary identified cancer anatomical sites.

Primary anatomical site	Number of incidences	Percentage
Lung	27	40.9%
Colon	6	9.1%
Breast	5	7.6%
Brain	3	4.6%
Skin	3	4.6%
Unknown Primary	3	4.6%
Bile Duct	2	3.0%
Gastroesophageal Junction	2	3.0%
Pancreas	2	3.0%
Prostate	2	3.0%
Retroperitoneum	2	3.0%
Testicular	2	3.0%
Bone	1	1.5%
Endometrial	1	1.5%
Ovarian	1	1.5%
Peridural	1	1.5%
Thyroid	1	1.5%
Urethral	1	1.5%
Urothelial	1	1.5%

### Altered Genes and Mutations

In 66 Foundation Medicine^®^ tests, 107 different mutations were found. The most common Foundation Medicine^®^ mutations identified were: TP53 (n = 31, 10.9%), CDKN2A (n = 19, 6.7%), KRAS (n = 16, 5.6%) and APC (n = 9, 3.2%). The median number of mutations found per patient was 3. By tumor location site, the median number of mutations found was 8.5 for gastroesophageal junction cancer, five for brain, breast and skin cancer, four for lung cancer, 3.5 for cancer of unknown primary and three for colon and pancreatic cancer. A summary of mutations identified are listed in **Table 4**. OncoKB is a system that maps identified mutations to drugs with variable levels of evidence (16). In our patient population, nine (12.5%) patients had a level 1 identified mutation; eight (11%) patients had a level 2 identified mutation; nine (12.5%) patients had a level 3a identified mutation; 19 (26.4%) patients had a level 1 &/or 2 &/or 3a mutation identified. A full list of all the mutations and their OncoKB score is outlined in **Tables 5**, **6**.

**Table 4:** Summary of oncogenic mutations identified in FM report.

Oncogenic mutation	Number of incidences	Percentage
TP53	31	10.9%
CDKN2A	19	6.7%
KRAS	16	5.6%
APC	9	3.2%
EGFR	7	2.5%
PIK3CA	7	2.5%
TERT	7	2.5%
MLL	7	2.5%
MYC	6	2.1%
SMAD4	6	2.1%
STK11	5	1.8%
MDM2	5	1.8%
BRAF	4	1.4%
ERBB2	4	1.4%
ZNF	4	1.4%
PTEN	4	1.4%
RICTOR	4	1.4%
FRS2	4	1.4%

n = 3: BRCA1, GNAS, ATM, NOTCH, CDK6, FGF, CDK4, BAP1.

n = 2: NRAS, ERBB4, TOP2A, AUKRA, ARFK1, PTCH1, ARIDIA, FGF23, LRP1B, FAT1, ROS1, CRKL, GATA3, CCND1, CD274 (PD-L1), Jak2, PDCD1LG2 (PD-L2), RET fusion, NF1, PBRM1, CCND2, GLI1, MAP2K4, MDM4, MYCN, TSC1, AKT, NF2, SEDTD2, CCND3, NKX2.

n = 1: Ntrk1, BRCA2, Mcl1, AKT3, EMSY, PALB2, FAS, FBXW7, CDK12, CYLD, KIT, TBX3, RBM10, SPAT1, BRAD1, INPP4B, MSH, LZTR1, DD1T3, PGDFRA, DICER1, U1AF1, ARFP1, PPP2R1A, ATRX, CREBBP, IGF1R, PIK3C2B, RANBP2, PASK, FGFR, NUP, CCNE, KDM, MAP2K, PRKCI, TERC, VEGFA, AXINI, FOXP1, SMO, P114L, G1202R, GATA2, BCOR, CD36, CIC, SMARC4A, FLT4, NFKB1A.

**Table 5:** OncoKB levels of evidence for identified mutations^a,b,c^.

		Level of Evidence for Mutation Treatment																															
		Mutations																															
Oncologic site	Oncologic type	BRAF	BRAF v600E	BRCA	BRCA1	BRIP1	CDKN2A	CHEK	EGFR	EGFR (exon 19)	ERBB	ERBB2 v569E	FGFR3	G1202R	KIT	KRAS	KRAS G12A	KRAS G12C	KRAS G12D	KRAS G12V	KRAS G13D	KRAS K117N	KRAS Q61L	KRASG12D	NF1	NRAS	P114L	P14ARF	PIK3CA	PTEN	RET	ROS1	TP53
Brain	Glioblastoma						4		4				4																				
	Astrocytoma								4																								
Breast	Lobular carcinoma				2																									4			
	Ductal carcinoma				2																								1, 3a	4			
Colon	Adenocarcinoma										2								4, R1		4, R1		4, R1			R1							
Gastroesophageal junction	Adenocarcinoma						4																							4			
Lung	Squamous Cell						4						4									4						4					
	Adenocarcinoma		1				4			1	2, 3a	2, 3a		1, R2			4	3a, 4	4	4					4					4	1, 2, 3a	1	
Pancreas	Adenocarcinoma						4						4			4												4					4
Peridural	Myxoid liposarcoma																										4						
Prostate	Adenocarcinoma					1																											
Retroperitoneum	Sarcoma						4																										
Skin	Melanoma						4						4		4																		
Testicular	Adenocarcinoma						4																										
Thyroid	Papillary Tall Cell Variant	4					4						4																				
Unknown Primary	Adenocarcinoma						4																										
Urethral	Squamous Cell						4																										

^a^Mutations may have multiple treatments with varying levels of evidence. ^b^Levels of evidence: 1—FDA approved, 2—Standard care, 3—Clinical evidence, 4—Biological evidence, R1/R2—Resistance. ^c^Mutations are reported as they were in the Foundation Medicine report, therefore further clarification (e.g. subtype of KRAS mutation) is not always possible.

**Table 6:** Mutations identified with OncoKB actionable level 1, 2 or 3.

Mutation^a^	OncoKB actionable level(s)
BRAF v600E	1
BRCA	2
BRCA1	2
BRIP1	1
CHEK	1
EGFR (EXON 19)	1
ERBB	2
ERBB2	2 & 3a
ERBB2 v569E	2 & 3a
G1202R	1
KIT	2
KRAS G12C	3a
PIK3CA	1 & 3a
RET	1, 2, & 3a
RET FUSION	1, 2, & 3a
ROS1	1

a Mutations are reported as they were in the Foundation Medicine report, therefore further clarification (e.g. subtype of KRAS mutation) is not always possible.

b Mutations may have multiple treatments with varying levels of evidence.

### Clinical Outcomes

Eight of 66 (=12%) patients had actionable mutations identified that led to a direct change in therapy, and those eight patients received 12 therapies directly related to the Foundation Medicine^®^ results (six (75%) patients had one Foundation Medicine^®^ result directed therapy, two (25%) patients had three Foundation Medicine^®^ result directed therapies. See **Table 7**).

**Table 7:** Summary of patients were Foundation Medicine® testing resulted in a direct change in treatment.

Sex	Age at diagnosis	Primary cancer site	Histological tumor type	Targeted mutation	Which line of treatment was target treatment(s)	Target treatment(s)	Response to target treatment	Vital status
Female	69	Lung	Adenocarcinoma	ROS-1	First	Crizotinib	PD	Alive
Male	63	Urethral	Papillary Urothelial Transitional Cell Carcinoma	FGFR3	Third	Pazopanib	PD	Dead
				TSC1	Fourth	Everolimus	SD	
				FGFR3	Fifth	Pazopanib	PD	
Female	39	Lung	Adenocarcinoma	ERBB2	Fifth	Trastuzumab	Indeterminable	Dead
Male	56	Lung	Adenocarcinoma	AKT3 Amplification	Fourth	Everolimus	PD	Dead
Male	23	Lung	Adenocarcinoma	ROS-1	Third	Crizotinib	PR	Alive
					Fourth	Lorlatinib	PR	
					Fifth	Cabozantinib	PD	
Female	46	Lung	Adenocarcinoma	ERBB2 v569E	Second	Afatinib	SD	Alive
Female	56	Endometrial	Stromal Sarcoma	STK11	Fourth	Everolimus	PD	Dead
Female	67	Lung	Adenocarcinoma	BRAF V600E	Second	Dabrafenib and Trametinib	PR	Alive

Among the 12 Foundation Medicine^®^ directed therapies, the clinical benefit rate was 5 which is 42% (those therapies that result in stable disease, partial response, or complete response). In our cohort of patients, the best response to a Foundation Medicine^®^ directed therapy was partial response which was found three times (25%). Six of the eight patients (=75%) who had their treatment modified due to FM^®^ testing had lung cancer.

The median survival from the date of diagnosis to death/last follow up in all patients is 34.7 months (IQR = 20.2–49.4 months).

The median survival from the date that Foundation Medicine^®^ test was performed to the date of death/last known follow up in all patients is 16.8 months (IQR = 4.9–25.4 months).

Among the patients where Foundation Medicine^®^ testing led to a direct change in therapy, the median survival from the date that Foundation Medicine^®^ test was performed to the date of death/last known follow up is 22.9 months (IQR = 11.5–30.3 months).

Among the patients where Foundation Medicine^®^ testing did not lead to a direct change in therapy, the median survival from the date that Foundation Medicine^®^ was test performed to the date of death/last known follow up is 16.5 months (IQR = 3.5–25.0 months).

## Discussion

### Summary of Results

Foundation Medicine NGS testing can identify a myriad of mutations in any given malignancy, an increasing number of which are actionable with novel therapies. In this series we found that eight patients (12%) had treatment altered based on their FM test result. Further, using the OncoKB classification, there were 10 level 1 mutations and eight level 2 mutations identified. The median survival of these patients with stages 3 and 4 disease was 22.9 months in patients with actionable mutations and 16.5 months in those with no actionable mutations. This impressive median survival was in spite of not many actionable mutations being found. There is likely a selection pressure, as patients with the most aggressive cancers would have less likely been able to access Foundation Medicine^®^ testing. The majority of cases in which Foundation Medicine^®^ testing lead to direct treatment options were lung cancer. Since the time that these reports were sent, some of these mutations would now be identified on routine screening-such as ROS-1 and BRAF. With these cases removed, only five patients would have actionable mutations, based on Foundation Medicine^®^ testing alone. The most common mutation identified was TP53, which is in line with prior research which reports TP53 as the most common mutation in cancer (17).

### Compare and Contrast our Results to Previous Studies

First, to compare the most common mutations found. A study on rare and refractory cancers, found that the most common mutations identified were TP53 (46%), followed by RAS/RAF/APK(45%) (8). A study on advanced solid tumors using FoundationOne CDx panel found TP53 (65%) followed by PIK3CA (19%) were the most common mutation identified (18). A final study using FoundationOne CDx testing found the most common mutations identified were KRAS (94%) followed by TP53 (76%) (19). In our study, the most common mutation was also TP53 (47%), but the second most common mutation identified in our study was CDKN2A (29%). Our study showed a median age of patients to be 56 years, which is similar to a study on hematological cancers sent for comprehensive genetic profiling, whom had a mean age of 54 years (10) but slightly dissimilar to the median age of 62 years found in patients with advanced solid tumors sent for FoundationOne CDx panel (18).

### Limitations

Methodological limitations of this study include single center review and small sample size thereby reducing the power of these results. Furthermore, since the period of study included, on-site mutational testing has become more comprehensive. ROS1 mutations are now tested for routinely and many centers have on-site NGS, or easier access to NGS through research protocols. These advances could reduce the potential benefit of private sample testing such as that offered by Foundation Medicine^®^. In our study, 10 patients were excluded due to problems with the sample to be tested. It is known that providing insufficient tissue is the primary reason for failure with next generation sequencing (20). Liquid biopsies provide a more heterogeneous sample and may be an alternative to better ensure that samples are not excluded (20). Thus, the results of this analysis may differ with tissue *vs.* liquid biopsies.

### Future Directions

This study focused on the demographics of the patients sent for Foundation Medicine^®^ testing, and their mutations found, as well as the treatment given based on this, and overall survival of all patients. This study was unique, and in addition to mutations found, the treatment outcomes and long-term survival were outlined. This study was not specific to one cancer, but a majority of actionable mutations were from lung adenocarcinoma cases. Future studies can focus on adenocarcinoma cases only, to see if there are certain clinical features that suggest a patient is more likely to benefit from Foundation Medicine^®^ testing, compared to others. Additionally, cost analysis studies can also be done to determine the effectiveness of foundation medicine testing, in finding new viable treatment options for patients.

Furthermore, many patients in our study had been identified with KRAS mutations (n = 16), five of which had G12C mutations. Current research on target therapies such as AMG 510 and MRTX849 have recently reported clinical efficacy in treating G12C mutation positive solid-type tumors (21), so while these patients did not have actionable therapy at the time, this is an example of the fast pace of drug discovery in the current cancer treatment era. Therefore, further studies on the clinical benefit of mutational study could be done as new target therapies become available.

## Conclusions

In conclusion, FM testing has not been routine in this center. Further, while multiple mutations are discovered only a small proportion were actionable mutations, of which some are now part of routine practice anyway. NGS testing is likely to become more widespread, but this research suggests its true clinical impact will be restricted to a minority of patients.

## Data Availability Statement

The raw data supporting the conclusions of this article will be made available by the authors, without undue reservation.

## Author Contributions

MM: Creation of project, data extraction and analysis, and manuscript writing. DK: Creation of project, data extraction and analysis, and manuscript writing. PW-P: Creation of project, and manuscript writing and editing. AA: Manuscript writing and editing. BL: Procurement of genetic testing data. Manuscript writing and editing. LM: Data extraction. All authors contributed to the article and approved the submitted version.

## Conflict of Interest

The authors declare that the research was conducted in the absence of any commercial or financial relationships that could be construed as a potential conflict of interest.

## Publisher’s Note

All claims expressed in this article are solely those of the authors and do not necessarily represent those of their affiliated organizations, or those of the publisher, the editors and the reviewers. Any product that may be evaluated in this article, or claim that may be made by its manufacturer, is not guaranteed or endorsed by the publisher.
